# Optimising Green Pressurised Liquid Extraction and Sustainability Assessment of Carotenoid-Rich Extracts from *Daucus carota* L. Pomace

**DOI:** 10.3390/foods14213740

**Published:** 2025-10-31

**Authors:** Lidia Favaretto, Stefania Pagliari, Ciro Cannavacciuolo, Luca Campone, Massimo Labra

**Affiliations:** Department of Biotechnology and Biosciences, University of Milano-Bicocca, 20126 Milan, Italy; l.favaretto@campus.unimib.it (L.F.); stefania.pagliari@unimib.it (S.P.); c.cannavacciuolo93@gmail.com (C.C.)

**Keywords:** Pressurised Liquid Extraction, *Daucus carota*, carrot juice by-products, carotenoids, circular economy, response surface design optimisation

## Abstract

The increasing food waste generated along the food chain should be considered as a source of high-value compounds, with the aim of improving the circularity of productions. In this study, carrot pomace, the major by-product of carrot juice processing, was used as a source of carotenoids. For the valorisation of this by-product, different non-conventional extraction methods of carotenoids such as Ultrasound-Assisted Extraction (UAE) and Pressurised Liquid Extraction (PLE) have been developed. For the latter, the main parameters influencing the extraction have been optimised using a multivariate response surface design. Compared with previous reports, this study advances the current knowledge by using only food-grade ethanol/water mixtures as solvents and by combining the optimisation of carotenoid recovery with the measurement of energy consumption to evaluate process efficiency. Moreover, the sustainability of the extraction was quantitatively assessed using the AGREEprep metric, providing a more integrated and environmentally sound strategy for the valorisation of *Daucus carota* L. pomace.

## 1. Introduction

Fruit and vegetables present severe losses in the food chain due to their perishable nature. According to FAO estimates, more than 20% of global fruit and vegetable production is lost between harvest and distribution [1]. Throughout the industrial processing of vegetables and fruits, serious amounts of by-products are generated in the form of non-edible parts, such as peels and pomace, which can still be refined into valuable products [2]. Among these, the production of carrot juice shows a significant loss rate, with up to 50% of the weight of the raw material being discarded in the form of pomace [3,4]. However, carrot pomace still contains large amounts of biologically active compounds [5]. Carrot (*Daucus carota* subsp. sativus) is a widely cultivated root vegetable plant, available in several colours, shapes, and sizes [6]. According to Eurostat data, in 2022, the European Union (EU) produced approximately 4.4 million tonnes of carrots. Carrots are valued for their high content of fibre, carotenoids, polyphenols, and antioxidants, making them a functional food [7,8,9]. Carotenoids in foods are commonly classified into carotenes and xanthophylls, pigments that give an attractive red or yellow colour and contribute to food quality [10]. They, especially β-carotene, are active mainly as a precursor of vitamin A but have other interesting biological effects such as their antioxidant property that helps protect against oxidative stress, supports the immune system, and reduces the risk of degenerative diseases such as cancer and cardiovascular and eye disorders [11,12,13,14]. The demand for carotenoids and apocarotenoids is rapidly increasing as more clinical trials reveal their health and medicinal benefits, and it is estimated that the worldwide carotenoid market will hit over USD 2 billion in the coming years [3,15]. To satisfy this demand, by-products such as carrots pomace could be valorised through the development of sustainable techniques for the extraction of carotenoids, together with other high-value bioactive components [16]. The selective extraction of some components from a solid matrix employing appropriate solvents is a known operation. In the case of polar compounds, water is certainly the most effective solvent, but apolar compounds should be extracted using a suitable organic solvent. Carotenoids as a group are extremely hydrophobic and mostly show little or no solubility in water. They are, however, soluble in organic solvents, which are therefore used in the traditional extraction methods [17]. The main drawback associated with this choice is the huge use of solvent and potential toxicity, especially when the resulting product is intended for human consumption. Therefore, the selection of a proper solvent and extraction technique plays a key role in the development of a technically and economically sustainable process.

Previous research has explored PLE and UAE for carotenoid recovery from carrot by-products and other sources, such as algae. Such research has mainly focused on defining and optimising the parameters that influence the extraction process to improve yield alone [18,19,20]. This study differs significantly in that it combines optimising carotenoid yield with directly measuring energy consumption as an indicator of sustainability. This study uses the AGREEprep eco-compatibility metric to assess the entire extraction process quantitatively. This further strengthens the valorisation of agro-industrial waste in the context of the circular economy. Therefore, it is necessary and highly beneficial to employ sustainable processes with minimal cost and environmental impact for the efficient and selective extraction of metabolites from carrot by-product [21,22]. Pressurised Liquid Extraction (PLE) stands out among various extraction methods for agricultural by-products due to its high efficiency compared with traditional techniques. PLE utilizes a liquid solvent at elevated temperature and pressure, enhancing the extraction efficiency of target compounds from the matrix. The combined effects of pressure and temperature improve mass transfer rates by reducing solvent surface tension and viscosity [23,24,25]. This dual action enhances analyte solubility, facilitating solvent penetration into the matrix. PLE offers several advantages over conventional methods like maceration, distillation, and Soxhlet extraction, including reduced energy consumption, improved waste management, shorter production times, cost effectiveness, enhanced consumer safety and health, regulatory compliance, and preservation of bioactive compounds in the matrix [23,25]. In this study, a Pressurised Liquid Extraction method was employed to recover carotenoids from pomace as a carrot juice by-product. Initially, an analytical procedure based on ultra-pressure liquid chromatography (UPLC) coupled with high-resolution mass spectrometry (HRMS) was developed to assess the chemical composition of the extract. Furthermore, recognising that PLE is influenced by various factors such as temperature, number of cycles, extraction time, and solvent composition, a response surface design was employed to identify the experimental parameters that maximise the recovery of α-carotene and β-carotene and reduce energy consumption. Under optimised extraction conditions, the developed PLE method was evaluated using the greenness index to confirm its sustainability and improved efficiency compared with conventional approaches.

## 2. Materials and Methods

### 2.1. Standards and Materials

The following mass-grade solvents were used for mass analysis: acetonitrile (MeCN), methanol (MeOH), isopropyl alcohol (IPA), water (H_2_O), and formic acid (HCOOH), which were provided by Romil (Cambridge, MA, USA). The analytical-grade solvents hexane (Hex), methanol (MeOH), ethanol (EtOH), ethyl acetate (AcOEt), and acetone were supplied by Sigma-Aldrich (Milan, Italy). Water was purified using a Milli-Q system (Millipore, Bedford, MA, USA). α-Carotene and β-carotene analytical standards were purchased from Merck KGaA (Darmstadt, Germany).

### 2.2. Samples

Fresh carrots (*Daucus carota* L., origin Italy) were purchased from several national supermarket chains located in different regions of Italy. To ensure a representative and homogeneous sample, multiple aliquots (several kilograms) from different geographical origins and production batches were combined before processing. The fresh roots were washed with distilled water, sliced manually with a stainless-steel knife, and extracted using an extractor for juice production. After juice extraction, the carrot pomace was stored at −80 °C overnight and subsequently freeze-dried in a laboratory-scale freeze-dryer (Benchtop pro, Steroglass, Perugia, Italy). The lyophilised matrix was further blended using an IKA M 20 Universal mill, 225 W (IKA-Werke GmbH & Co., Staufen, Germany), and the resulting powder was sieved with W.S. Tyler Test Sieves (W.S. Tyler, 8570 Tyler Boulevard, Mentor, OH, USA) to obtain 150–300 μm sized particles. The matrix was stored in sealed bags in a dark and dry environment until use.

### 2.3. Optimisation of PLE Conditions by an Experimental Design

The extractions were carried out on a Dionex ASE300 (Dionex, Sunnyvale, CA, USA). Briefly, 500 mg of dried material was put into a 10 mL stainless-steel extraction cell for the extraction operation. The extraction cell’s empty area was filled with 4 mm solid glass beads (Sigma-Aldrich, Milan, Italy), and a paper filter (Whatman n°1) was inserted at the bottom. Preliminary tests were performed at a pressure of 1000 psi for 5 cycles and 5 min static time with different solvents (ethanol 100%, ethyl acetate 100%, and acetone 100%) and temperatures (70 °C, 100 °C, and 130 °C) to identify the best solvent and temperature range to be used in the experimental design. The extracts were collected in a glass vial (60 mL); 500 µL was collected for the quali-quantitative analysis, and the solvent was later evaporated in a vacuum evaporator at 30 °C to calculate the extraction yield. To select the best extraction conditions of the PLE, a central composite experimental design (CCD) was performed using Statgraphic Centurion XVI Version 16.1 (Rockville, MD, USA). The effects of four independent factors on extraction efficiency and weight yield were studied through an experimental design. The range for each factor was selected by preliminary experiments. In particular, a response surface Box–Behnken design 2-factor interaction was carried out considering four variables at three different levels (low, medium, and high): extraction temperature at 60, 80, and 100 °C; number of cycles 2, 4, and 6; composition of solvent (% H_2_O/EtOH) at 0, 10, and 20%; and static time 3, 5, and 7 min. The considered response variables were the quantity of α-carotene and β-carotene recovered (mg/g of matrix, to be maximised) and energy consumption during the extraction process (kW/h, to be minimised), measured using an energy monitor (KTEM01EU model, KETOTEK, Xiamen, China). A total of 27 experiments (27 points of the factorial design, 3 centre points, and 12 degrees of freedom) were performed in a randomised run. To obtain the parameters of the statistical models, data from the CCD were submitted to regression analysis using least-square regression methods. Analysis of variance (ANOVA) was used to determine the statistical significance of independent variables’ (A, B, C, and D) contributions and their first-order interaction. The standardised Pareto chart was used to analyse the influence of each component on the response variable, and the response surfaces of the mathematical models were created. The answers obtained from the statistical analysis were fitted to a second-degree model capable of considering the individual parameter interactions together with their quadratic relationships.

### 2.4. Qualitative and Quantitative Analysis of Carotenoids and Polar Fraction of Extract by HPLC-UV/Vis

The quali-quantitative analysis of carotenoid extracts was carried out by HPLC-UV/Vis on a 1260 Infinity II LC System (Agilent Technologies, Santa Clara, CA, USA, 2018) equipped with a binary pump (G-1312A) and a UV detector (G-1314A). The extracts were reconstituted in acetonitrile/methanol/isopropyl alcohol (85/10/5 *v*/*v*) and separated on a C18 column (100 Å, 2,6 μm, 100 × 2.1 mm, KINETEX) at a flow rate of 0.4 mL min^−1^; solvent A was acetonitrile, solvent B methanol, and solvent C isopropyl alcohol. An isocratic elution was performed within 20 min at an 85:10:5 *v*/*v* ratio of solvents A/B/C. Carotenoids were detected at a wavelength of 450 nm with a DAD array detector and confirmed using α-carotene and β-carotene standards.

The qualitative analysis of carotenoids was carried out using the external standard approach. Calibration curves for α-carotene and β-carotene were established across a concentration range of 0.1–10 mg mL^−1^ to determine their levels in the extract. The calibration curves exhibited strong linearity, with correlation coefficients (R^2^) of 0.9994 for α-carotene and 0.9991 for β-carotene.

### 2.5. Statistical Analysis

All experiments were performed in triplicate, and the results are presented as average ± standard deviation. ANOVA was used to compare the means, while Turkey’s test was used to assess the statistically significant difference among extraction conditions using JMP 14 software. A *p*-value of ≤0.05 was considered significant.

## 3. Results and Discussion

This work aimed not only to optimise carotenoid recovery but also to assess the sustainability of the PLE process. Compared with previous reports on PLE or UAE of carrot pomace [19,20], the present study combines yield optimisation with direct energy measurement and quantitative greenness evaluation (AGREEprep Calculator). This dual objective provides a clearer, more realistic perspective on how extraction performance compromises with process sustainability.

### 3.1. Evaluation of Chemical Composition of Carrot By-Product

The chemical composition of the carrot by-product could be quite variable and is influenced by many factors such as its origin and the processing to which it has been subjected [26]. For this reason, to investigate the chemical composition of the carrot by-products, a preliminary Pressurised Liquid Extraction under mild conditions (70 °C, two extraction cycles, and 80% EtOH) was performed. Identification of compounds was achieved by using all chemical information: retention times (Tr), UV/vis signals, accurate mass, molecular formula, MS/MS spectra, and reference standards whenever available, combined with chemo-taxonomic databases. UPLC-HRMS/MS analysis of the PLE hydroalcoholic extract revealed two main peaks corresponding to the carotenoids α- and β-carotene. Traces of polyphenols were also identified, including chlorogenic acid, p-cumaroylquinic acid, 3-O-feruloylquinic acid, di-caffeoylquinic acid, and isorhamnetin-3-O-glucoside. As the phenolic fraction was found to be of little interest and was mainly transferred during juice extraction, subsequent steps focused on the carotenoid fraction to enhance the value of carrot pulp.

### 3.2. Optimisation of Carotenoid Extraction by PLE

#### 3.2.1. Preliminary Selection of Solvent Composition and Temperature

**Figure 1:**
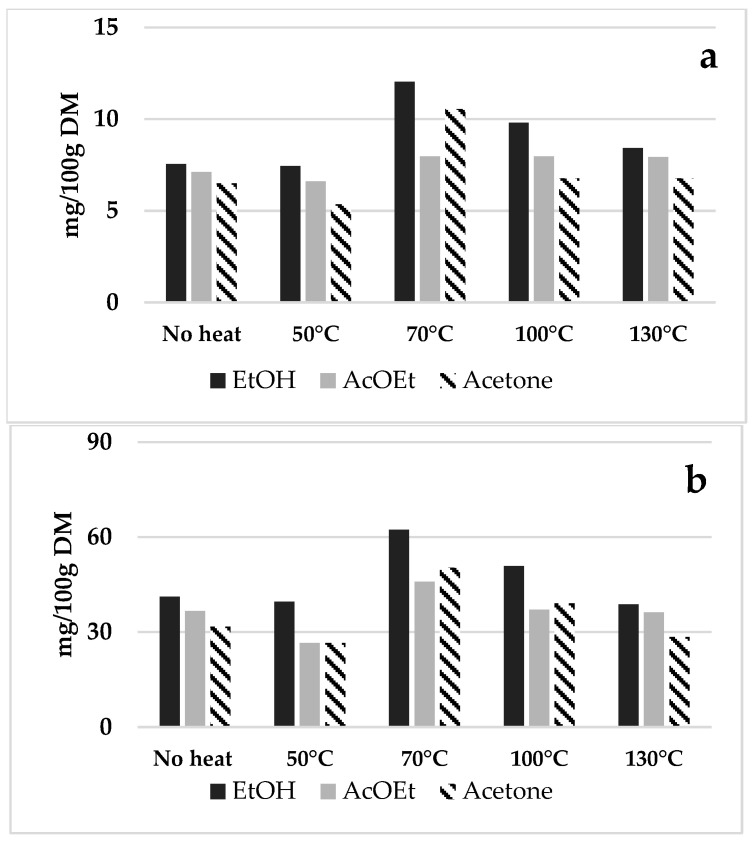
Effect of the temperature and different solvents on the recovery of α-carotene (**a**) and β-carotene (**b**) using three different solvents: ethanol (EtOH), ethyl acetate (AcOEt), and acetone. The results are expressed as mg/100 g DM in PLE extracts.

Given that the contribution of the phenolic fraction did not seem to be of interest in terms of either qualitative or quantitative composition, we decided to concentrate our attention on the recovery optimisation of the carotenoids. For these reasons, the extraction conditions for the sustainable recovery of carotenoids were optimised using a chemometric approach. As commonly reported in the literature, temperature and solvent composition are considered the most critical parameters in the PLE process [27]. Consequently, a preliminary experiment was conducted to determine the appropriate temperature range for use in the experimental design. The temperature was systematically increased from 25 to 130 °C, with monitoring of the carotenoid content using three solvents: ethanol, ethyl acetate, and acetone. The other extraction parameters of the PLE system were maintained at mild conditions (two cycles, 5 min static time). The effect of PLE temperature with different solvents on the extraction efficiency of carotenoids is shown in Figure 1.

The results indicate a similar trend for both carotenoids, with a correlation observed between the increase in PLE temperature and extraction efficiency up to 70 °C. However, beyond this temperature, the recovery of target compounds begins to decline, likely due to the degradation of these carotenoids. As also reported in the literature, carotenoids are thermosensitive molecules, with many degrading at temperatures exceeding 100 °C [28,29]. Based on these findings, the chemometric optimisation was conducted within a temperature range of 60–100 °C. Regarding solvent composition, ethanol demonstrated better extraction efficiency than ethyl acetate and acetone for both carotenoids. Therefore, given its higher extraction efficiency combined with its characteristics being generally recognised as safe (GRAS), ethanol was chosen for further experiments.

#### 3.2.2. Response Surface Design of PLE Process

Once ethanol was selected as a promising solvent for the extraction and preliminary experiments defined the temperature range, a chemometric approach was carried out to find the best experimental conditions and optimise the extraction process, using experimental design methods. As is well known, PLE is a technique influenced simultaneously by several parameters, including temperature, static time, solvent composition, pressure, and cycles [25]. Therefore, selecting the optimal parameters is crucial for achieving the highest extraction efficiency. For this purpose, a Box–Behnken two-factor interaction design was utilised to explore the impact of four independent variables (temperature, number of cycles, static time, and percentage of ethanol) on the dependent variables, which are the extraction efficiency of the two carotenoids and the reduction of energy consumption. Table 1 presents the experimental conditions for each run and the corresponding experimental values of the responses (α-carotene, β-carotene, and energy consumption) under different experimental conditions.

**Figure 2:**
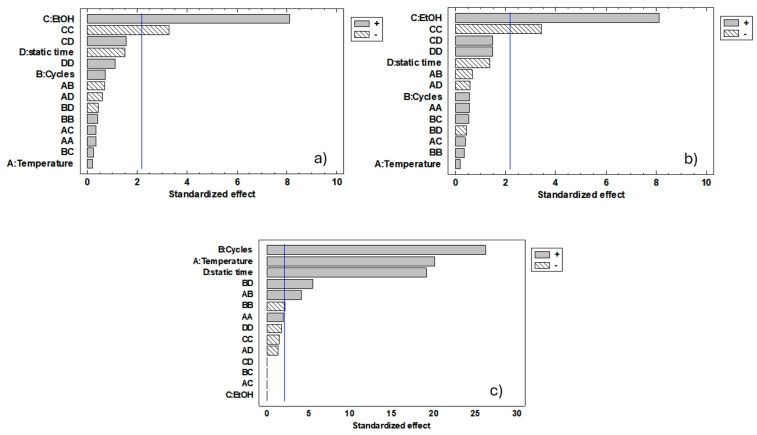
The standardised Pareto chart of response variables (α–carotene (**a**), β–carotene (**b**), and energy consumption (**c**)) shows the estimated effects and interactions of each independent factor in the Box–Behnken design model in order of decreasing significance. Bars beyond the vertical line are statistically significant at a 95% confidence level; the positive effects are shown in grey bars and negative effects in dashed bars.

The statistical significance of the response variables was assessed through standardised Pareto charts for each experimental factor (Figure 2), and the statistical analysis is reported in Table S1. Based on the obtained results, the model showed a good correlation (R^2^ ≅ 88–99%), indicating a good prediction of the model for all the response variables considered.

**Figure 3:**
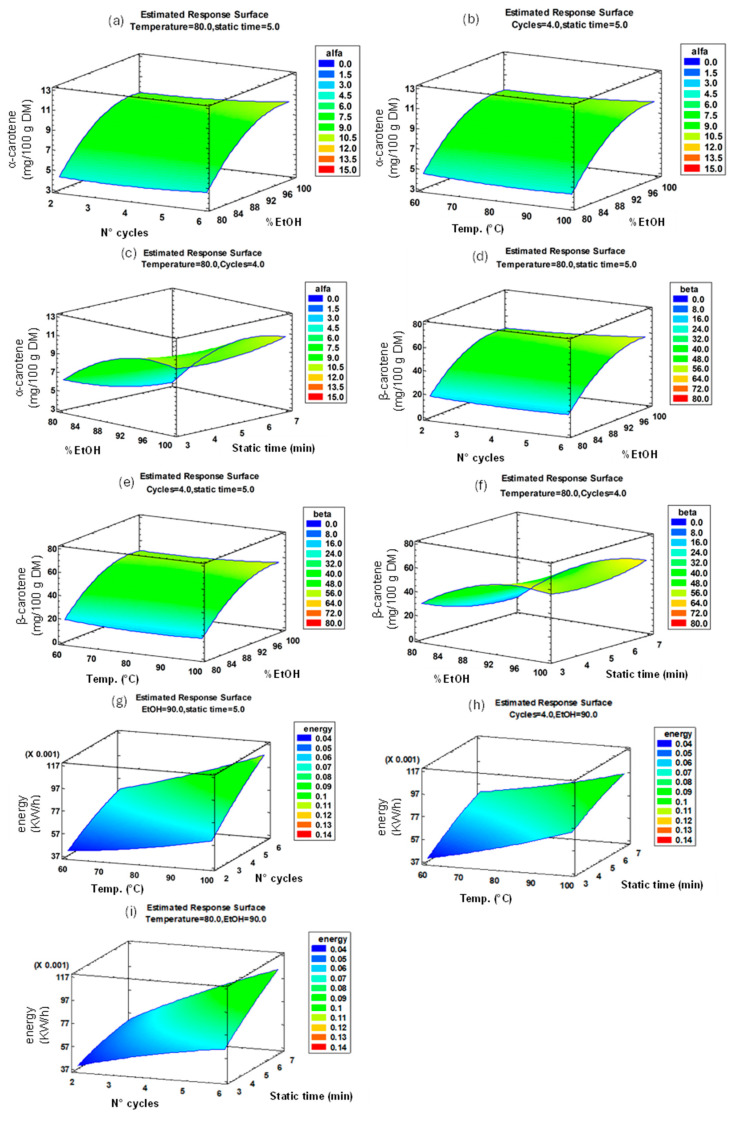
Response surface graphs showing the effects of the following combinations on carotenoid yield: %EtOH vs. number of cycles (**a**,**d**); %EtOH vs. temperature (**b**,**e**); and %EtOH vs. static time (**c**,**f**). Also shown are the effects of the following combinations on energy consumption: temperature vs. number of cycles (**g**); temperature vs. static time (**h**); and number of cycles vs. static time (**i**) on energy consumption.

The joint analysis of the Pareto charts (Figure 2a,b) and the response surfaces (Figure 3a–f) shows that the percentage of ethanol (EtOH) is most influential parameter in the model, positively contributing to the two response variables α-carotene and β-carotene. In fact, increasing the percentage of EtOH in the extraction solvent, there is an increase in the recovery efficiency. This behaviour can be explained by the properties of the solvents used in the extraction process. Ethanol, being a polar protic solvent (less polar than water), decreases solvent polarity and enhances the solubility of hydrophobic carotenoids compared with a hydroalcoholic solvent.

The other independent variables (temperature, static time, and number of cycles) did not significantly affect the efficiency of carotenoid extraction. Therefore, when considering only the optimisation of carotenoid recovery, the optimised extraction conditions should be as follows: EtOH 95%, temperature 86 °C, six cycles, and static time 3 min (optimised A). This combination aligns well with the conditions reported in the literature when using the same extraction technique. Similar extraction efficiencies were achieved under comparable parameters (60 °C, 5 min of static time, and five extraction cycles) [19]. However, temperature, number of cycles, and static time are the only independent factors that significantly affect energy consumption (Figure 2c) and, consequently, the cost of the entire extraction process. There is a linear increase in energy consumption as temperature, the number of cycles, and residence time increase (Figure 3g–i). This suggests that the energy requirements of the extraction process are mainly determined by thermodynamic and operating conditions rather than by the nature of the solvent. To make the process more sustainable in terms of costs and environmental impact, the optimised conditions were recalculated to maximize the extraction of α-carotene and β-carotene and at same time reduce the energy consumption: EtOH 100%, temperature 82 °C, cycles 2, static time 7 min (optimised B), with a desirability index optimum of 91.53%.

### 3.3. Quantitative Analysis of PLE Extract

Quantification of α- and β-carotene in the PLE extract obtained under optimised conditions (optimised B) was conducted using UPLC-UV with an external standard approach, revealing concentrations of 11.28 ± 0.71 and 67.42 ± 2.33 mg/100 g DM, respectively. These values are in good agreement with those predicted by the regression models developed for the three response variables, confirming their reliability as predictors.

Furthermore, comparing the optimised B with the predicted values for optimised A revealed no statistically significant differences in carotenoid concentration but confirmed a strong reduction in energy consumption (Table 2), indicating that optimised B is the most efficient in terms of extraction yield and energy sustainability of the PLE process. A comparison with the existing literature shows that the optimised PLE conditions used in this study allow for a greater recovery of carotenoids than that with UAE. For example, Umair et al. reported a yield of 14.89 ± 0.40 μg/g of β-carotene and 31.82 ± 0.55 μg/g of total carotenoids from carrot pomace. Meanwhile, Mantiniotou et al. obtained 137 μg/g of β-carotene equivalents/g of sample [20,30]. The methodology proposed in this study allows for improved extraction efficiency while maintaining the use of ethanol, a safe solvent suitable for food applications. Non-polar solvents, such as acetone, hexane, or ethyl acetate, are known to increase carotenoid recovery (from 980 to 1728 μg/g DW) [16]; however, their higher toxicity severely limits their use in food applications, so they were not considered in this study.

These findings highlight the potential of the PLE extract as a valuable source of carotenoids, which could be a promising ingredient for incorporation into functional foods, dietary supplements, and nutraceutical applications. Due to their conjugated double-bond structure, carotenoids are susceptible to oxidation, as well as photo- and thermal degradation [31,32]. Several studies have evaluated the stability of carotenoids during storage and concluded that refrigerated temperatures improve stability [33,34]. To overcome these limitations, different strategies have been proposed, including microencapsulation through spray-drying or freeze-drying techniques [35]. These processes have already been applied to reduce oxidation and extend the shelf life of carotenoid-rich extracts during storage [35]. Such approaches could therefore enhance the stability of the extract and make it more suitable for future industrial applications.

### 3.4. Greenness of PLE Optimised Process

**Figure 4:**
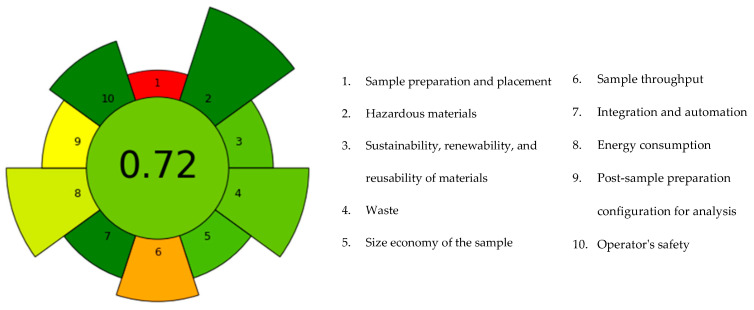
Evaluation of the extraction protocol using AGREEprep—Analytical GREEnness Metric for Sample Preparation [36].

In the field of bioactive compound extraction, there is an increasing emphasis on environmentally friendly techniques. Therefore, assessing the sustainability of sample preparation methods is crucial for developing green processes. The sustainability of the proposed method was evaluated using the AGREEprep calculator, which is based on the ten principles of Green Sample Preparation (GSP) [36]. The 10 criteria demonstrate different levels of importance in terms of their impact on sustainability (the larger the segment, the greater the criterion’s impact). The contribution of each criterion was maintained as set in the analyser. This tool provides a normalised value between 0 and 1; a value closer to 1 indicates a process that is more environmentally sustainable. In the case of the PLE extract obtained under optimised conditions B, a score of 0.72 was achieved (Figure 4).

A score above 0.7 is generally considered to be indicative of good sustainability compared with conventional methods, which often show lower values [37]. Analysis of the individual criteria reveals the most critical and influential factors in the sustainability of the process and helps identify possible changes to improve it. The analysis showed that the criteria with the most impact in our analysis are numbers 1, 6, and 9. Specifically, transporting the sample and processing it at a site other than the collection site increases resource consumption due to logistics. However, this criterion is not directly correlated with extraction parameters and was therefore left as a smaller segment. The second criterion was the limited number of samples that can be processed per hour (criterion 6). However, the latter could be addressed through further process optimisation, such as automatisation or implementation of continuous flow extractors. Despite these limitations, the proposed PLE approach shows good potential from an industrial perspective. It is based exclusively on GRAS solvents, operates under moderate conditions, and requires short extraction times. These factors are compatible with currently available semi-continuous PLE systems. Although the AGREEprep score (0.72) indicates favourable environmental performance on a laboratory scale, industrial feasibility requires a thorough techno-economic assessment (TEA). A preliminary analysis, based on our lab-scale data, highlights key parameters for scalability such as energy consumption per kg of pomace, solvent throughput, and solvent recovery efficiency, which directly impacts both operational costs and environmental footprint. To further enhance the sustainability of the process, future efforts should focus on strategies such as continuous extraction systems, solvent recycling by distillation, and optimising thermal integration. Although a comprehensive TEA remains a necessary step, current results suggest that the process is particularly well suited for high-value products such as carotenoid concentrates, where solvent reuse and energy optimisation can ensure economic viability [38,39].

Finally, the greenness index is also strongly influenced by the impact of the analytical step (high-performance liquid chromatography) used for quantitative analysis (criterion 9). This step is necessary just during the optimisation of the extraction process. Overall, the results obtained suggest that this method is a promising solution for sustainable extraction with potential applications in the agri-food, cosmetics, and pharmaceutical sectors.

## 4. Conclusions

This study demonstrates that extraction using pressurised liquids (PLE) is a valid and eco-friendly alternative for recovering carotenoids from carrot juice by-products, adding value to a waste material that would otherwise be discarded and thus aligning with the principles of the circular economy. Using a multivariate response surface design, key parameters were optimised to maximise the recovery of α-carotene and β-carotene, while minimising environmental impact as evidenced by a greenness index of 0.72. Additionally, the exhausted matrix could be repurposed for applications such as animal feed or biofuel production, further contributing to waste minimisation. The findings of this study offer a comprehensive and sustainable strategy for the extraction of high-value bioactive compounds from agricultural by-products. Future work should focus on scaling up the optimised extraction process and evaluating the stability and bioavailability of the recovered carotenoids during storage. For potential industrial applications, a crucial future perspective involves a systematic study of the stability and degradation kinetics of the extracted carotenoids. Their susceptibility to factors like temperature, light, and oxygen necessitates the development of advanced stabilisation strategies. In this context, encapsulation techniques using nano-carriers, such as polysaccharides, proteins, lipids, or more-complex systems like nanoliposomes and solid lipid nanoparticles represent a promising approach to protect these compounds and maintain their functionality during storage. Overall, the research provides a robust framework for sustainable extraction processes that can be applied not only to carrot by-products but also to other similar matrices, promoting circular economy through the recovery of high-value-added compounds.

## Figures and Tables

**Table 1:** Experimental conditions and corresponding quantitative values of the three selected response variables (α-carotene, β-carotene, energy consumption) obtained from the 27 runs of the response surface design.

Run	Temperature	Cycles	EtOH	Static Time	α-Carotene	β-Carotene	Energy Consumption
	°C	n°	%	min	mg/100 g DM	mg/100 g DM	kW/h
1	80	4	80	7	3.06	11.3	0.078
2	80	4	90	5	8.34	41.6	0.072
3	80	6	100	5	11.08	61.8	0.084
4	80	6	80	5	4.18	11.8	0.085
5	80	6	90	3	9.41	52.6	0.063
6	80	4	100	3	10.67	60.1	0.051
7	100	4	90	3	10.30	57.0	0.074
8	60	2	90	5	8.51	46.6	0.037
9	60	4	90	7	9.19	51.2	0.073
10	60	6	90	5	9.65	53.6	0.069
11	80	2	80	5	3.5	11.5	0.048
12	100	4	80	5	3.07	11.4	0.086
13	80	4	100	7	9.20	50.5	0.079
14	100	2	90	5	9.84	54.2	0.055
15	80	2	90	7	9.38	52.3	0.056
16	100	4	90	7	9.60	53.8	0.098
17	80	2	100	5	9.80	53.2	0.049
18	60	4	100	5	10.17	55.0	0.058
19	80	4	90	5	9.63	52.4	0.070
20	80	2	90	3	8.64	47.7	0.040
21	80	6	90	7	9.07	50.4	0.106
22	100	4	100	5	9.42	51.5	0.087
23	100	6	90	5	9.29	50.5	0.109
24	60	4	90	3	8.42	45.4	0.041
25	80	4	80	3	8.29	44.3	0.052
26	80	4	90	5	7.92	43.6	0.072
27	60	4	80	5	4.68	21.2	0.057

**Table 2:** Comparison of α-carotene and β-carotene recovery levels and energy consumption between two extraction scenarios: optimised A, maximising carotenoid yield, and optimised B, minimising energy consumption.

	Predicted Optimised A	Predicted Optimised B	Optimised B
α-Carotene (mg/100 g DM)	11.20	11.49	11.28 ± 0.71
β-Carotene (mg/100 g DM)	63.74	65.59	67.42 ± 2.33
Energy consumption (kW/h)	0.070	0.052	0.054 ± 0.02

## Data Availability

The original contributions presented in this study are included in the article/Supplementary Materials. Further inquiries can be directed to the corresponding authors.

## Supplementary Materials

The following supporting information can be downloaded at https://www.mdpi.com/article/10.3390/foods14213740/s1: Table S1: Analysis of variance of the regression mode.

## Abbreviations

The following abbreviations are used in this manuscript:

MeCN	Acetonitrile
ANOVA	Analysis of variance
CCD	Central composite experimental design
EtOH	Ethanol
AcOEt	Ethyl acetate
HCOOH	Formic acid
GSP	Green sample preparation
Hex	Hexane
HRMS	High-resolution mass spectrometry
IPA	Isopropyl alcohol
MeOH	Methanol
PLE	Pressurised liquid extraction
GRAS	Recognised as safe
UPLC	Ultra-pressure liquid chromatography
H_2_O	Water
TEA	Techno-economic assessment
