# Supplementary material for: Optimising Green Pressurised Liquid Extraction and Sustainability Assessment of Carotenoid-Rich Extracts from Daucus carota L. Pomace
Source: Foods. 2025 Oct 31;14(21):3740. doi: 10.3390/foods14213740 (PMC12609071; doi:10.3390/foods14213740)
Supplement: Supplementary file 1 [file foods-14-03740-s001.zip › foods-3905982-supplementary.pdf]

Table S1: Analysis of variance of the regression model

	Sum of squares			Mean squeres		
	**alpha**	**beta**	**energy**	**alpha**	**beta**	**energy**
A:Temperature	0.0675	2.43	0.00258133	0.0675	2.43	0.002581
B:Cycle	0.755008	19.2533	0.00437008	0.755008	19.2533	0.00437
C:EtOH	93.8561	4055.36	0.0	93.8561	4055.36	0.0
D:static time	3.23441	117.813	0.00232408	3.23441	117.813	0.002324
A^2	0.18253	18.6668	0.000025037	0.18253	18.6668	2.5E-05
B^2	0.238008	7.94898	3.22315E-05	0.238008	7.94898	3.22E-05
C^2	15.2776	718.169	1.33704E-05	15.2776	718.169	1.34E-05
AB	0.714025	28.6225	0.00011025	0.714025	28.6225	0.00011
AC	0.1849	9.9225	0.0	0.1849	9.9225	0.0
AD	0.540225	20.25	0.00001225	0.540225	20.25	1.23E-05
BC	0.09	17.2225	0.0	0.09	17.2225	0.0
BD	0.2916	11.56	0.000196	0.2916	11.56	0.000196
CD	3.5344	136.89	0.0	3.5344	136.89	0.0
DD	1.74041	134.447	2.04537E-05	1.74041	134.447	2.05E-05
Total error	17.0958	738.695	7.65833E-05	1.42465	61.5579	6.38E-06
Total (corr.)	145.821	6460.61	0.00978763			
R^2	88.2762	88.5662	99,2175			
Adj R^2	74.5984	75.2267	98,3047			

	F-value			P-value		
	**alpha**	**beta**	**energy**	**alpha**	**beta**	**energy**
A:Temperature	0.05	0.04	404.47	0.8313	0.8458	**0.0000**[a]
B:Cycle	0.53	0.31	684.76	0.4806	0.5863	**0.0000**[a]
C:EtOH	65.88	65.88	0.00	**0.0000**[a]	**0.0000**[a]	1.0000
D:static time	2.27	1.91	364.17	0.1577	0.1917	**0.0000**[a]
A^2	0.13	0.30	3.92	0.7266	0.5920	0.0710
B^2	0.17	0.13	5.05	0.6899	0.7256	**0.0442**[a]
C^2	10.72	11.67	2.10	**0.0066**[a]	**0.0051**[a]	0.1734
AB	0.50	0.46	17.28	0.4925	0.5083	**0.0013**[a]
AC	0.13	0.16	0.00	0.7249	0.6951	1.0000
AD	0.38	0.33	1.92	0.5495	0.5769	0.1911
BC	0.06	0.28	0.00	0.8058	0.6065	1.0000
BD	0.20	0.19	30.71	0.6590	0.6724	**0.0001**[a]
CD	2.48	2.22	0.00	0.1412	0.1617	1.0000
DD	1.22	2.18	3.20	0.2907	0.1652	0.0987
Total error						
Total (corr.)						
R^2						
Adj R^2						

R^2 = Quadratic correlation coefficient. [a] Significant ($p < 0.05$).
